# Exam performance of different admission quotas in the first part of the state examination in medicine: a cross-sectional study

**DOI:** 10.1186/s12909-020-02069-6

**Published:** 2020-05-25

**Authors:** Alex Mommert, Josefin Wagner, Jana Jünger, Jürgen Westermann

**Affiliations:** 1 Division of Study and Teaching, Faculty of Medicine, University of Lübeck, Ratzeburger Allee 160, House 2, 23562 Lübeck, Germany; 2 The German National Institute for state examinations in Medicine, Pharmacy and Psychotherapy, Mainz, Germany; 3 Institute of Anatomy, University of Lübeck, Lübeck, Germany

**Keywords:** Medical school selection, Admission, Interviews, Exam performance, Admission quotas

## Abstract

**Background:**

Most medical students in Germany are admitted via selection procedures, which are adjusted to the demands of the universities. At Lübeck medical school, scores from interviews that measure non-academic skills and pre-university GPAs are summed to arrive at an admission decision. This article seeks to illuminate the effectiveness of this selection procedure in comparison to other non-selected student groups.

**Methods:**

Quota information and exam results from the first federal exam were linked for students admitted to Lübeck medical school between 2012 and 2015 (*N* = 655). Five different student groups (university-specific selection quota, pre-university GPA quota, waiting time quota, ex-ante quota and foreign students) were compared regarding exam attempts, written and oral grades, temporal continuity and examination success in the standard study period.

**Results:**

While the pre-university GPA quota outperformed all other quotas regarding written and oral grades, it did not differ from the selection quota regarding exam attempts, temporal continuity and examination success in the standard study period. Students in the waiting time and ex-ante quotas performed inferior by comparison. The results of foreign students were the most problematic.

**Conclusion:**

Students selected by the university show high temporal continuity and examination success. These results, and possible advantages in physician eligibility, argue for the utilisation of non-academic skills for admission.

## Background

The German study place allocation for medicine introduced in 2004 follows a complex procedure [1]. First, a certain proportion of places is reserved for a prioritised (ex-ante) quota. This quota consists of i.a. foreign students, military personnel and hardship cases. Next, 20% of the remaining places are assigned to two quotas each: the grade point average (GPA) quota and the waiting time (WT) quota, that mainly rely on a single criterion for submission. Finally, 60% of the remaining places are assigned to a university-specific selection quota, for which universities may use specific criteria.

The liberty of the universities to define their selection methods has led to numerous differing allocation procedures. Pre-selection of candidates can rely on pu-GPA, location preference, bonus systems or some of these criteria combined. Only less than one-third of German public medical schools forego a preselection procedure [2]. Regarding admission itself, the procedures become even more diverse. Most of the German universities utilise pu-GPA in combination with other criteria, such as interview scores, ability or knowledge tests, grades in specific subjects (e.g. Biology) or completed vocational training. Only a small percentage of universities exclusively use the pu-GPA for the university-specific admission.

The pu-GPA is a well-known predictor of academic success in both medicine [3, 4] and other disciplines [5, 6]. Cognitive ability, diligence and persistence may be underlying factors of the GPA and certainly are important characteristics for studies in medicine. However, modern interpretations of physician roles emphasise skills apart from academic abilities. The CanMEDS framework, for example, stresses the importance of communicative, collaborative and leadership skills [7]. The role of such skills is further emphasised by the Word Health Organization that identified them as essential prerequisites for effective interprofessional care [8, 9]. Consequently, recent expert reports in Germany recommend social skills to be taught and evaluated in the course of the medical curriculum [10, 11]. Taken together, there is an increasing emphasis on social skills in the education of future physicians. To assess those skills, various methods are used, including interviews in different formats and degrees of structure [12, 13], personal statements [14], reference letters [15], situational judgement tests [16], and tests of emotional intelligence [17]. Yet, no other method has received as much attention as interviews [18–20]. Thus, the potential weaknesses of interviews (e.g. rater-bias, social desirability effects) and corresponding counter measures have been studied in depth [21]. To ensure their fairness and prognostic validity, interviews should be reasonably structured, use biographical and situational questions and utilise an unbiased, competence-based assessment [18, 22]. This includes an appropriate training of the interviewers, albeit it may further increase the costs of such a selection procedure. However, even cost-intensive admission procedures for medical studies may pay off [23].

Lübeck medical school (LMS) has set up a selection procedure that aims to meet the indicated demands for future physicians. For preselection, candidates may improve their pu-GPA based on a bonus system. Bonuses are granted for successful participation in an ability test (Test for Medical Studies—the most widely used ability test in Germany), completed vocational training and extracurricular achievements (e.g. voluntary service or a prize in the German contest for young scientists). Based on the improved pu-GPA, LMS invites 240 candidates for selection interviews, twice as many as can be placed that year. These interviews are conducted by 12 panels, each comprising three members, two faculty members and one student. Panel members participate in a general instruction session and especially new panel members are encouraged to participate in an interview-training workshop in which two mock interviews are conducted to familiarize them with interview techniques, question types and general conduct. For the interviews, the panel members are provided with a standardised interview guide that gives examples of situational and behavioural questions [18]. Panel members are encouraged to probe topics and responses in a flexible manner.

While the interview itself is semi-structured, LMS utilises a fully structured procedure for assessment [22]. Five primary dimensions that have to be rated individually by panel members address motivation, knowledge about the course of study, social engagement, (self-)reflection and communication. Each dimension contains five items that are rated on a five-point rating scale ranging from 0 (not at all) to 4 (entirely). Thus, a maximum rating of 100 can be obtained. The interview score is calculated as the mean of the three panel members’ ratings and transformed to fit a 30-point-scale.^1^ Pu-GPAs are transformed to fit a 31-point-scale and both components are then summed to arrive at a candidate’s final score.

The selection procedure at LMS was set up to meet the demands of contemporary accounts of physicians’ roles and expert reports by taking social skills into account. The remote aim is to educate physicians who will meet future job demands [24]. However, the selection procedure is also quite popular among the interested parties. Interviews are among the most popular university selection methods in Germany [25]. Candidates especially value the opportunity to introduce themselves personally. The LMS selection procedure consistently generates very high levels of acceptance among the candidates. Since 2012, more than 1500 interviews have been conducted; yet, not a single candidate contested the interview-based admission decision. The quality of the selection procedure has been consistently monitored since its introduction.

The scope of the current article is to illuminate the effectiveness of the LMS selection procedure by comparing the exam performance of students of different admission quotas. Although the aim of the LMS selection procedure is not to promote top exam performance (see above), a heightened dropout or failure rate in that quota would be problematic. Accordingly, a study from the Netherlands found that the non-academic criteria of a selection procedure had an influence on dropout rates: students who met the non-academic selection criteria (e.g. via experience in health care jobs) showed stronger adherence in their studies than students who did not [26]. Thus, we will examine exam attempts, written and oral grades, temporal continuity and exam success in the standard study period as different indicators of exam performance to evaluate the LMS selection procedure.

## Methods

### General methodology

We performed a cross-sectional study with quota information as predictors and four different indicators of exam performance as outcome measures (see below). The First Part of the State Examination in Medicine (M1) is the first of three federal exams that must be taken by German medical students to graduate (except for some of those enrolled in model courses). The M1 exam is conducted by the German National Institute for state examinations in Medicine, Pharmacy and Psychotherapy (IMPP). Quota information of all students who started their studies between 2012 and 2015, and were still enrolled at the LMS in February 2018, was linked with the M1 exam results of the IMPP. If a student took the exam more than once, data of the last exam attempt was used. In addition to the main quota information (Selection, GPA, WT and Ex ante quota) provided by LMS, we were able to identify foreign students using the IMPP data. All students with a foreign high-school diploma were designated to the foreign students quota.

### Dependent measures

We focused on the M1 exam results because this exam constitutes a standardised federal examination conducted by the vast majority of German medical students. Therefore, four aspects of exam performance were considered: exam attempts, written and oral grades, temporal continuity and examination success in the standard study period. The preliminary analysis illuminated the rate of students that attempted the M1 exam in the different quotas and thus qualified the results of the subsequent main analysis. The analysis of exam attempts also served as a first indicator of study progress. Written and oral grades served as standard performance indicators. Temporal continuity served as the indicator of study progress in the main analysis – now restricted to those students who attempted the exam in the examined timeframe. Finally, examination success in the standard study period constituted a holistic performance indicator that integrated information on performance (exam pass or fail) and study progress. Only passed exams within the standard study period were considered a success.

### Study sample

The dataset for the main analysis comprised data of 655 students, including 397 students from the LMS selection quota (60.6%), 98 students from the GPA quota (15%), 87 students from the WT quota (13.3%), 31 students from the ex-ante quota (4.7%) and 28 foreign students (4.3%). For 14 students (2.1%), no quota information was available. The latter cases were excluded from the analyses and will not be discussed in this article.

**Fig. 1:**
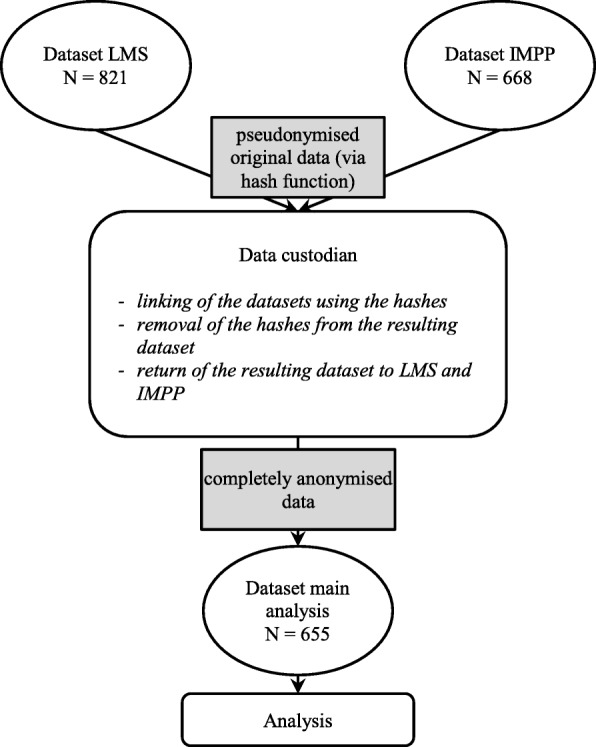
Linkage of data between Lübeck Medical School (LMS) and the German National Institute for state examinations in Medicine, Pharmacy and Psychotherapy (IMPP)

As part of the quality management of LMS, all analyses were performed using fully anonymised data. Data linkage was conducted by an expert third party data custodian (see Fig. 1) to ensure privacy. As part of the linkage, all individual identification features were omitted from the dataset. The procedure used conforms to the federal state data protection act.

For the main analysis, all cases were used for which quota and exam information was available. The main analysis was thus restricted to those students who took the M1 exam in the observed time period. Hence, we additionally conducted a preliminary analysis to observe the rate of cases for which this linkage was feasible. This analysis served as a proxy and illuminated the rate of students that attempted the M1 exam in the different quotas.

### Statistical analysis

The binary dependent variables exam attempts (0 – not attempted, 1 – attempted) and examination success in the standard study period (0 – not successful, 1 – successful) were analysed using logistic regression with admission quota as categorical predictor variable. The continuous dependent variables written and oral grades as well as temporal continuity were analysed using one-way analysis of variance (ANOVA) with admission quota as independent variable. Additionally, we present descriptive data of our sample (sex, pu-GPA and age).

The significance level for all statistical tests was set at *p* = 0.05. As we were particularly interested in the comparison of the different admission quotas, we performed multiple comparisons using post hoc contrasts for all dependent variables. For these multiple comparisons, we used the Benjamini-Hochberg procedure to control for accruing false discovery rates [27]. Rather than adjusting *p*-values overall, this procedure computes a list of critical values against which the unadjusted *p*-values are set [27]. In our tables, we present the overall test statistic and indicate the significance of the different comparisons using superscripts. Values with the same superscript do not differ significantly, while different superscripts indicate significant differences between these values.

There was no missing data for any of the analysed variables and all analyses were conducted using in IBM SPSS Version 21.

## Results

### Exam attempts

Overall, a large majority of the students took the M1 exam (91.4%). However, considerably strong differences existed between the quotas (see Table 1). While 94.7% of the students in the selection quota took the exam, this was the case for only 80.6% of the students in the waiting time quota (Odds ratio = 4.36).^2^

**Table 1:** Attempts to take the exam by admission quota

	Quota					
	Selection	GPA	WT	Ex ante	Foreign	χ^2^(3671)
Proportion that attempted the exam	94.7%^a^	90.7%^a,b^	80.6%^c^	86.1%^b, c^	−^1^	20.31^***^

^***^p < .001

^a, b, c^ Different superscripts indicate significant differences

^1^ Figure undeterminable

Selection: students selected by Lübeck medical school; *GPA* students admitted on basis of their pre-university GPA; *WT* waiting time; *Ex-ante* prioritised students; *Foreign* foreign students

### Main analysis

The proportion of female students was highest in the GPA (70.4%) and the selection quota (68.0%). Foreign students (60.7%) and students in the WT quota (57.5%) were also predominantly female. In the ex-ante quota, the sex ratio was almost balanced (51.6% female).

Students in the GPA quota displayed the best grades (*M* = 1.02, *SD* = 0.05) in their university entrance certificate,^3^ followed by students in the selection quota (*M* = 1.46, *SD* = 0.24). Behind them were students of the ex-ante quota (*M* = 1.80, *SD* = 0.47) and the WT quota (*M* = 2.46, *SD* = 0.36). Regarding the grades of foreign students, no information was available.

At the time the exam was taken, students in the GPA quota (*M* = 21.8 years, *SD* = 1.4) and selection quota (*M* = 22.6 years, *SD* = 1.9) were markedly younger than foreign students (*M* = 24.6 years, *SD* = 5.2) and students in the ex-ante quota (*M* = 25.1 years, *SD* = 4.4). Students in the WT quota displayed the highest mean age (*M* = 29.3 years, *SD* = 2.4).

Regarding the performance on the written part of the M1 exam, students in the GPA quota performed better than students from the selection quota (see Table 2); followed by students in the ex-ante and WT quota as well as foreign students. A similar pattern was observed for the oral part of the exam, with the difference that students in the WT quota performed slightly better than students in the ex-ante quota.

**Table 2:** Exam performance by admission quota

	Quota					
	Selection *M* (*SD*)	GPA *M* (*SD*)	WT *M* (*SD*)	Ex ante *M* (*SD*)	Foreign *M* (*SD*)	*F*(4, 636)
Grade written test	2.9 (0.8)^a^	2.2 (0.9)^b^	3.2 (0.9)^c^	3.0 (0.9)^a,c^	3.5 (0.9)^c^	22.24^***^
Grade oral test	2.3 (0.8)^a^	2.0 (0.8)^b^	2.6 (1.0)^c^	2.8 (0.8)^c^	3.0 (0.9)^c^	13.19^***^

^***^p < .001

^a, b, c^ Different superscripts within a row indicate significant differences

Selection: students selected by Lübeck medical school; *GPA* students admitted on basis of their pre-university GPA; *WT* waiting time; *Ex-ante* prioritised students; *Foreign* foreign students

**Fig. 2:**
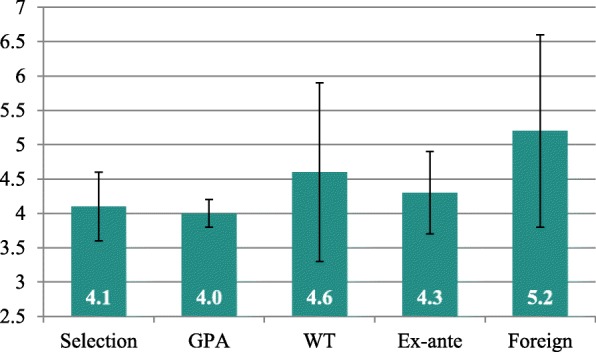
Semesters taken (mean) when attempting to take the exam by admission quota Whiskers represent ±1 standard deviation.

Regarding the temporal continuity of study—represented by the number of semesters studied when taking the M1 exam—there were large differences between the students of the different quotas, *F*(4, 636) = 24.83, *p* < .001 (see Fig. 2). Students in the selection, GPA and ex-ante quotas did not delay taking the exam and showed mean semester scores close to the practical minimum of four semesters. Students from the WT quota needed more than half a semester more before taking the exam, and foreign students needed more than a whole semester extra. The selection and GPA quotas differed significantly (post-hoc contrast tests using Benjamini-Hochberg procedure) from the WT quota and the foreign students, respectively. The contrast between the latter quotas was also significant, and there was a significant difference between the ex-ante quota and the foreign students.

Finally, we compared the passing rate in the standard study period (see Table 3). The data showed that students of the GPA and selection quotas ranked best regarding this criterion, and the foreign students exhibited the lowest passing rate.

**Table 3:** Exam success in the standard study period by admission quota

	Quota					
	Selection	GPA	WT	Ex ante	Foreign	χ^2^(4641)
Passing rate in the standard study period	88.7%^a^	92.2%^a^	70.1%^b,c^	80.6%^a,b^	46.4%^c^	45.36^***^

^***^p < .001

^a, b, c^ Different superscripts indicate significant differences

Selection: students selected by Lübeck medical school, *GPA* students admitted on basis of their pre-university GPA, *WT* waiting time, *Ex ante* prioritised students, *Foreign* foreign students

## Discussion

The scope of this study was to illuminate the effectiveness of the LMS selection procedure by comparing the exam performance of students in different admission quotas at LMS. Five different quotas were compared regarding exam attempts, written and oral grades, temporal continuity and examination success in the standard study period.

Students of the university-specific selection quota most reliably attempted the M1 exam. The original datasets were selected such that all of the included students should have attempted the exam, the last included cohort (freshmen in 2015) at least when studying in the standard period of study. Study delay, dropout or transfer to another university are possible reasons why students did not attempt to take the exam. Thus, the analysis reveals first evidence for potentially problematic study progress. The relevance of such interruptions at the individual and organisational level will be discussed in more detail below. Furthermore, the results of the preliminary analysis qualify the findings of the main analysis since the latter was restricted to those students who took the exam.

Students in the GPA quota outperformed students in the other quotas in the written and the oral part of the exam. Students in the selection quota were the next best performers, followed by students in the WT and ex-ante quota. Foreign students displayed the poorest grades in both parts of the exam. Interestingly, the difference between the GPA and selection quotas was markedly weaker in the oral part of the exam. This may be attributed to different demands; while the written part employs multiple choice questions and primarily aims at the reproduction of knowledge, the oral part also communicative skills and the application of knowledge comes into play. Likewise, this interpretation may also explain why WT students perform markedly better in the oral compared to the written part of the exam. Prior experiences of selection and WT students in medical or medical-related areas could be crucial for this finding. Our results are in line with recent findings [3, 4, 28] and underscore the aptitude of the pu-GPA as an admission criterion. Consequently, recent juridical changes in the German study place allocation procedure for medicine resulted in an expansion of the GPA quota from 20 to 30% [29].

Students of the selection and the GPA quota displayed the highest levels of temporal continuity, especially compared to WT and foreign students. A very similar pattern was observed for passed exams within the standard study period. That is, selection and GPA quota students were not only the most reliable groups regarding exam participation after the standard four semesters, they also displayed the highest proportions of examination success. The inferior pu-GPAs in the selection quota compared to the GPA quota do not give rise to significant differences in temporal continuity or passing rates between these student groups. Continuity and exam success in the WT quota were inferior by comparison; however, the performance of this group was by far less problematic than suggested in other publications [30].

Foreign students yielded the most problematic results in our analyses. This group achieved the poorest grades on the exam, displayed the lowest level of temporal continuity and only less than half of these students passed the exam within the standard study period. Foreign medical students in Germany are already known to perform poorly [31–33]. Undeniably, foreign students face numerous challenges, especially regarding language, integration, culture and finances [34]. This group seems to be in special need of support by their university to master these challenges. Fortunately, the problems of internationals have received heightened attention, and possibly promising support measures are currently being tested [35]. We hope that the measures implemented at LMS (e.g. special introductions and language courses) will improve the situation for this group.

In our analyses, we emphasise study progress, since it is an important indicator of study success. A student may only take the M1 exam if the requirements of the first four semesters are met. Furthermore, at the individual (student) level, other reasons may exist explaining why there are interruptions in the course of studies. Devoting time to activities apart from studies—be it a job, social or political engagement or pursuit of a hobby—have proven to affect study progress [36]. Delays in study progress, however, have been found to be linked to mental distress [37]. At the organisational (university) level, interruptions ultimately pose perils; accumulating interruptions or transfers may create additional teaching and administrative costs. Thus, although individually justified, interruptions in study progress might have a negative impact on those affected and potentially impose additional institutional costs. In this sense, the empirical findings regarding study progress are most beneficial in the selection and the GPA quota.

The university-specific selection quota performed quite well, but that group was outperformed by the GPA quota. Studies have shown, however, that non-academic selection procedures develop their predictive validity for the advanced, clinical stage [26, 38]. This might be due to personality differences that seem to exist between the students of different admission groups [39]. Future studies should focus on the advanced course of study and physician aptitude, where possible advantages of the selection quota are likely to come into play [40].

Unsurprisingly, taking non-academic skills into account does not seem to promote perfect examination scores. Attempts in Germany to further improve exam grades by selecting students with an additional academic test yielded disillusioning results. Not only were the scores of such tests unrelated to study progress, but students admitted on the basis of the pu-GPA and an additional test, in fact, exhibited worse grades compared to students admitted exclusively on the basis of the pu-GPA [41]. From our point of view, a focus on a selection procedure that aims to select the best future physicians is far more beneficial for society than a procedure that seeks to select excellent future students. We think that considering non-academic skills seems to be an important step in building the future physician workforce.

Compared to interviews, less costly procedures exist that allegedly measure non-academic skills more objectively. Thus far, however, validity of Situational Judgement Tests (SJTs) used in Germany is not satisfactory [2]. In addition, the measurement of psychosocial skills, with the help of SJTs, has proven trainable and even vulnerable to faking [42, 43]. Possible counter measures are only currently under investigation [44]. Regardless of the specific methodology, we strongly argue for the use of a valid measure of non-academic skills in addition to pu-GPA for student selection. This allows applicants to compensate for non-excellent grades, and thus broadens students’ access to medical training.

This study was the first to compare the exam performance of students in five different admission quotas at LMS. As such, it exhibits some limitations. First, we used an exploratory approach conducting post-hoc contrast tests. The accumulating false discovery rates were controlled Benjamini-Hochberg procedure [27, 45]. The second limitation is also methodological in nature: the observed groups differed considerably strongly in size. These differences are due to legal requirements [1] and would have persisted even with a bigger total sample. The sample size in our study was determined to meet the statistical requirements of the conducted analyses [46, 47]. Third, although the role of age and sex might be of interest for some readers, it was beyond our scope to investigate the causal relationship between these variables and the outcome measures. Finally, our results were obtained within the specific selection procedure at LMS and cannot be easily generalised to other contexts. With accumulating evidence regarding the importance of non-academic skills for study success [26, 38], however, we are confident that similar procedures will lead to similar results elsewhere.

## Conclusion

We argue for the use of a valid measure of non-academic skills for student selection. Considering non-academic skills as an admission criterion for medical students has two major societal benefits. First, since non-excellent grades can be compensated, it broadens the access to medical training. Second, it helps to meet contemporary demands in the training of future physicians. Selection interviews pose an appropriate method to achieve these benefits. Selected students might yield lower examination grades, but as we have shown, they display impeccable study progress and success. Thus, the benefits for society are not associated with drawbacks for the universities.

## Data Availability

The datasets used and analysed during the current study are available from the corresponding author on reasonable request.

## Abbreviations

GPA: Grade point average
IMPP: German National Institute for state examinations in Medicine, Pharmacy and Psychotherapy
LMS: Lübeck medical school
M1: First Part of the State Examination in Medicine
SJT: Situational judgement tests
pu-GPA: Pre-university grade point average
WT: Waiting time

## Footnotes

1 In this process, the interview score is corrected for panel strictness/leniency using z-standardisation.

2 The figure for foreign students is undeterminable for this analysis, since the respective quota-information was obtained from the IMPP-dataset.

3 Grading in Germany usually ranges between 1 (best possible grade) and 6 (worst possible grade).
