# Correction to: Exam performance of different admission quotas in the first part of the state examination in medicine: a cross-sectional study

**DOI:** 10.1186/s12909-020-02098-1

**Published:** 2020-06-25

**Authors:** Alex Mommert, Josefin Wagner, Jana Jünger, Jürgen Westermann

**Affiliations:** 1 Division of Study and Teaching, Faculty of Medicine, University of Lübeck, Ratzeburger Allee 160, House 2, 23562 Lübeck, Germany; 2 The German National Institute for state examinations in Medicine, Pharmacy and Psychotherapy, Mainz, Germany; 3 Institute of Anatomy, University of Lübeck, Lübeck, Germany

**Correction to: BMC Med Educ (2020) 20:169**

**https://doi.org/10.1186/s12909-020-02069-6**

Following publication of the original article [1], it was noted that due to a typesetting error, the Table 1 and Table 3 were processed incorrectly. The correct tables are given below.

**Table 1:** Attempts to take the exam by admission quota

	Quota					
	Selection	GPA	WT	Ex ante	Foreign	χ^2^(3, 671)
Proportion that attempted the exam	94.7%^a^	90.7%^a,b^	80.6%^c^	86.1%^b, c^	−1	20.31***

***p < .001

^a, b, c^Different superscripts indicate significant differences

^1^ Figure undeterminable

Selection: students selected by Lübeck medical school; GPA: students admitted on basis of their pre-university GPA; WT: waiting time; Ex-ante: prioritised students; Foreign: foreign students

**Table 3:** Exam success in the standard study period by admission quota

	Quota					
	Selection	GPA	WT	Ex ante	Foreign	χ^2^(4, 641)
Passing rate in the standard study period	88.7%^a^	92.2%^a^	70.1%^b,c^	80.6%^a,b^	46.4%^c^	45.36***

***p < .001

^a, b, c^Different superscripts indicate significant differences

Selection: students selected by Lübeck medical school, GPA: students admitted on basis of their pre-university GPA, WT: waiting time, Ex ante: prioritised students, Foreign: foreign students

Tables 1 and 3.

The original article has been corrected.
